# A time-series clustering analysis of postinduction blood pressure trajectories

**DOI:** 10.1038/s41598-025-33740-x

**Published:** 2026-01-16

**Authors:** Maxim Glebov, Maksim Katsin, Haim Berkenstadt, Dina Orkin, Yotam Portnoy, Adi Schuchami, Teddy Lazebnik

**Affiliations:** 1 Department of Anesthesiology, Sheba Medical Center, Ramat Gan, Israel; 2 Faculty of Medicine, Tel-Aviv University, Tel Aviv, Israel; 3 Department of Mathematics, Ariel University, Ariel, Israel; 4 Department of Information Systems, University of Haifa, Haifa, Israel; 5 Department of Computing, Jonkoping University, Jonkoping, Sweden

**Keywords:** Cardiology, Diseases, Health care, Medical research

## Abstract

Induction of general anesthesia is often associated with significant hemodynamic changes, particularly in blood pressure (BP). These early postinduction fluctuations can vary widely among patients and contribute to perioperative complications. Current clinical approaches to managing postinduction BP changes are largely reactive and may not fully account for individual variability. This study aimed to identify distinct patterns of mean arterial pressure (MAP) response during the first 10 min following induction of general anesthesia, using a time-series clustering approach. We conducted a retrospective cohort study of 17,645 adult patients undergoing non-cardiac, non-obstetric inpatient surgery under general anesthesia at a tertiary medical center. BP was measured at 1 min intervals using either invasive arterial lines (8.3% of cases) or standard non-invasive oscillometric cuffs. An unsupervised X-means clustering algorithm with dynamic time warping was applied to identify recurring MAP trajectory patterns. Patient demographics, comorbidities, anesthetic drug doses, and other perioperative characteristics were compared across clusters. Five distinct MAP trajectories were identified: Initial Decline—Plateau (31.8%), Gradual Moderate Decline (18.4%), Initial Decline—Recovery (7.5%), Gradual Severe Decline (29.6%), and Initial Decline—Low Plateau (12.7%). These patterns differed significantly in baseline MAP, comorbidity profiles and antihypertensive use, while differences in anesthetic agent doses were statistically but not clinically meaningful. Distinct postinduction BP trajectories were identified using a time-series clustering approach. These findings provide a framework for future validation in datasets with richer clinical context.

**Supplementary Information:**

The online version contains supplementary material available at 10.1038/s41598-025-33740-x.

## Introduction

Induction of general anesthesia (GA) is frequently associated with significant hemodynamic disturbances, particularly in blood pressure (BP), which can vary widely among patients^1^. Recent studies have shown that even short durations of hypotension are associated with adverse outcomes. For example, as little as one minute of exposure to mean arterial pressure (MAP) below 55 mmHg has been linked to an increased risk of acute kidney injury after noncardiac surgery^2^. Postoperative acute kidney injury in turn is associated with an eightfold increase in postoperative mortality^3^. These risks highlight the need for improved strategies to anticipate and manage BP changes during the induction phase.

Traditionally, anesthesiologists have relied on a combination of clinical experience and real-time monitoring to anticipate and manage postinduction BP changes. However, this approach is inherently reactive, addressing hemodynamic instability after it has already manifested, and it may not account for the wide spectrum of BP responses. Furthermore, this approach may fail to accommodate the wide spectrum of BP responses observed across different patient populations^4^. This variability underscores the need for a more proactive and individualized approach to managing perioperative hemodynamics.

While postinduction hypotension is a well-recognized phenomenon, most previous studies have relied on binary thresholds or single-point definitions (e.g., MAP < 65 mmHg) to describe it^5^. However, these approaches may not adequately capture the temporal variation and complexity of blood pressure responses during induction. In clinical practice, we observe substantial heterogeneity in the timing, magnitude, and shape of BP changes, suggesting that distinct patterns may exist. Based on this observation, we hypothesized that a limited number of reproducible postinduction MAP trajectories could be identified.

Advanced clustering techniques can reveal distinct patterns within extensive datasets, providing new perspectives on hemodynamic behavior during and immediately after GA induction^6^. Such approaches may not only enhance our understanding of BP changes during anesthesia induction but also support the development of personalized anesthetic management strategies.

In this study, we analyzed perioperative data from 17,645 patients undergoing various surgical procedures under GA. Our objective was to characterize distinct postinduction MAP trajectories in a large, real-world surgical cohort as a foundation for future research into trajectory-based risk stratification and individualized hemodynamic management.

## Materials and methods

### Study design and data collection

The study protocol of this retrospective observational study was approved by the Ethics Committee of Sheba Medical Center, Israel (SMC-D 0649–23, November 2, 2023). The committee waived the informed consent requirement. All analyses were performed in accordance with relevant guidelines and regulations. The study adhered to principles outlined in the STROBE guidelines for observational studies. Data were collected from the electronic health records (Chameleon, Elad Software Ltd., Tel Aviv, Israel) of all adult patients who underwent inpatient, non-obstetric, non-cardiac surgery under GA with intravenous induction at Sheba Medical Center between September 1, 2018, and September 1, 2023. All included surgeries were performed in the main inpatient operating theaters. We excluded patients undergoing ambulatory, outpatient, or day-case procedures, including minimally invasive surgeries conducted in dedicated satellite surgical units.

All included patients received propofol for induction of anesthesia, followed by maintenance with inhalational agents. Patients who underwent total intravenous anesthesia (TIVA) for maintenance were excluded from the analysis.

The following exclusion criteria were applied: (1) patients who arrived intubated to the operating room; (2) those with mechanical circulatory support devices; and (3) those who received vasoactive drugs at any time prior to induction, during induction, or within the first 10 min following induction. We also excluded patients with missing baseline values or inadequate arterial blood pressure data, defined as measurements missing for at least two consecutive minutes. Blood pressure values classified as artifacts were excluded, including systolic arterial pressures ≥ 250 mmHg or ≤ 50 mmHg, and mean arterial pressures ≥ 200 mmHg or ≤ 20 mmHg.

Baseline MAP was defined as the final recorded blood pressure value—either invasive or non-invasive—obtained immediately prior to administration of induction agents. In patients without invasive arterial monitoring, BP was measured using standard non-invasive oscillometric cuffs. At our institution, the anesthesia monitoring system is configured to record non-invasive BP measurements at 1 min intervals during induction and for the first 10 min of anesthesia, as part of standard intraoperative care.

We focused on the first 10 min following induction to capture the immediate postinduction blood pressure fluctuations for clustering and trajectory analysis. Minimum alveolar concentration (MAC) values were recorded as the average over this 10-min postinduction period, representing early anesthetic exposure during maintenance.

The final dataset included a wide array of variables, including demographic information, preoperative comorbidities, intraoperative details, and anesthetic drug doses (Supplemental Material 1). All data processing and statistical analyses were conducted using Python (version 3.9). The time-series X-means clustering algorithm was implemented using the “tslearn” library (version 0.6.3)^7^.

### Data analysis

#### Clustering method

For clustering the blood pressure trajectories, we adopted the time series X-means method^8^,^9^. Each patient was represented by a vector of size 10, corresponding to the MAP during the first 10 min after induction, measured with one one-minute interval. Based on this representation, we chose the number of clusters (K) that we hypothesized were present in the data. Each cluster was represented by a vector indicating the “average” patient in a group, called the centroid. The first K centroids were randomly selected from the dataset. Subsequently, in an iterative process, the groups were improved using two steps. First, for each patient in the dataset, we calculated the distance from the data point to each of the K centroids and assigned each patient to the nearest centroid (i.e., with the smallest distance) using the dynamic time warp metric with the Sakoe-Chiba bounding, which restricts the dynamic time wrap metric to a fixed range of temporal difference; here we set the bound for two minutes. This results in K clusters that are based on the closest centroid. Following the first step, we recalculated the centroids in the K clusters. The new centroid of a given cluster was the average (mean) of all data points assigned to that cluster. These two steps were repeated until no changes were observed in the clusters between two successive iterations. Importantly, as the true number of clusters was not known a priori, we repeated the above procedure for K values ranging from 1 to 10. This range was selected to include any potentially clinically meaningful number of distinct blood pressure trajectory patterns. We considered values greater than 10 to be clinically unreasonable, as further subdivision would likely yield overly granular clusters with limited interpretability or practical relevance in anesthetic practice. For each of these cases, we calculated the quality of the clusters using the Bayesian Information Criterion (BIC)^10^, which considers the goodness of fit of the model and penalizes models with more clusters to avoid overfitting, following standard practices. The K value that gave the highest BIC value indicated the optimal number of clusters. To facilitate transparency and reproducibility, the full Python code used for data preprocessing, trajectory clustering, and visualization is provided in the Supplemental Material 2.

#### Cohort analysis

Patient characteristics were summarized and compared using appropriate descriptive and inferential statistics. Continuous variables were assessed for normality using the Shapiro–Wilk test, as well as visual inspections of histograms and Q–Q plots. Variables with a normal distribution were reported as mean ± standard deviation (SD), while non-normally distributed variables were reported as median and interquartile range (IQR), expressed as actual ranges (Q1–Q3) for clarity. Categorical variables were presented as proportions (%). Comparisons of patient characteristics between different clusters were performed using one-way analysis of variance (ANOVA) for normally distributed continuous variables, and the Kruskal–Wallis test for non-normally distributed continuous variables. Additionally, categorical variables across the five MAP trajectory groups were compared using the Chi-squared test. All statistical tests were two-sided, and statistical significance was set to* p* < 0.05.

## Results

### Patient characteristics

**Fig. 1:**
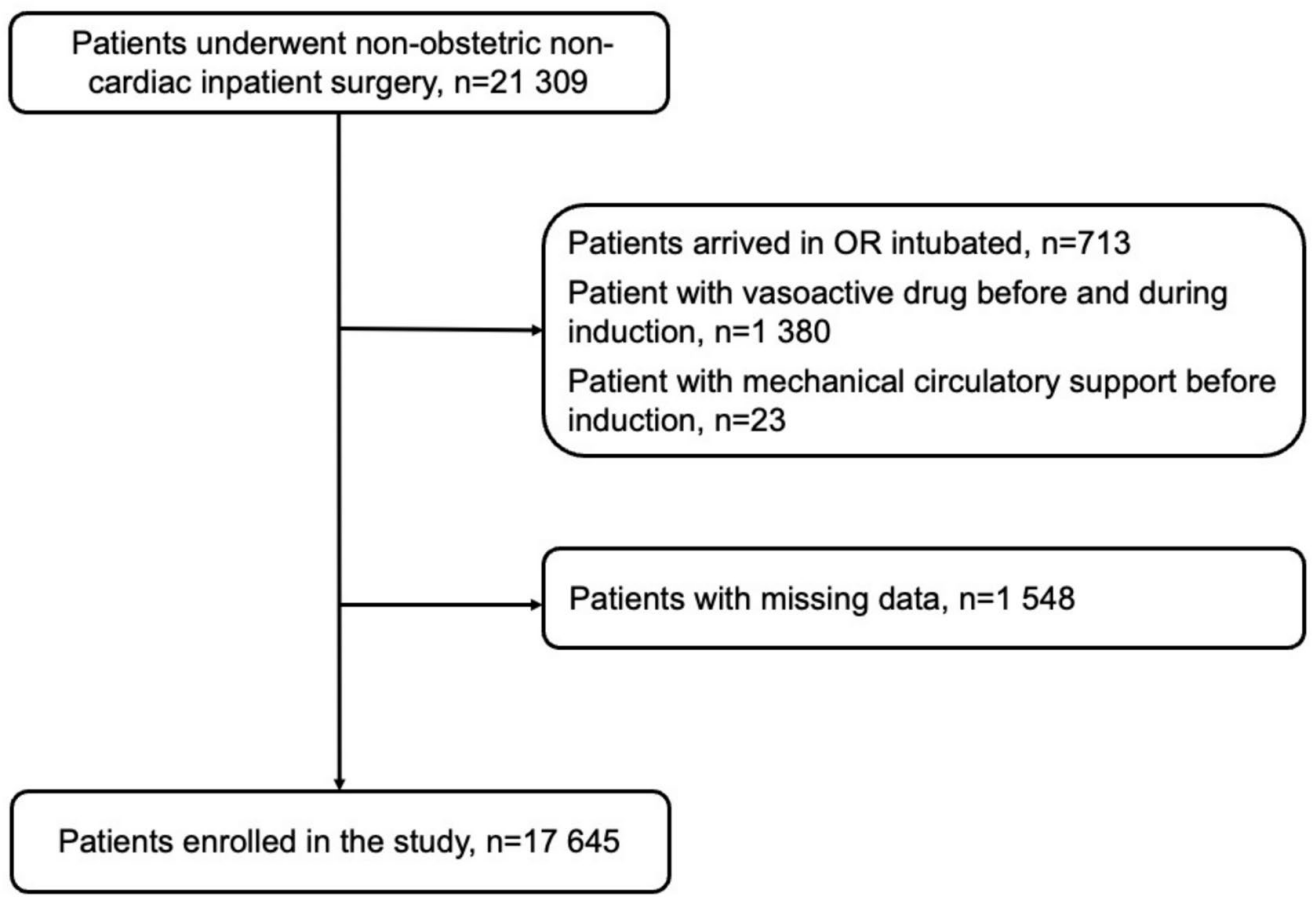
Patient enrollment flow chart.

Patient selection is reported in the flow chart (Fig. 1). A total of 17,645 patients were included in the final analysis. The median patient age was 48.3 [31.4 – 65.2] years. The study included 9,437 men (53.5%) and 8,208 women (46.5%). The majority of patients had ASA-PS class II or III [6,233 (35.3%) and 6,789 (38.5%)], respectively. Of the total cohort, 14,720 (83,4%) patients underwent elective surgery. A complete breakdown of patients by operating department is provided in Table S1.

The mean pre-induction MAP was 103 ± 18 mmHg. In this study, invasive BP monitoring, involving the insertion of a pre-induction arterial line, was carried out in 1473 (8.3%) of cases. Additionally, in 1511 (8.6%) of cases, invasive BP monitoring commenced only after induction had concluded. The cohort characteristics are presented in Table 1.

**Table 1:** Patients’ characteristics.

Variable type	Variable	Value
Demographic parameters	Age, years, median [Q1-Q3]	48.3 [34.2 – 68.0]
	Male, *n* (%)	9437 (53.5%)
	BMI, kg/m2, mean ± SD	26.9 ± 6.1
Surgical/anesthetic factors	Elective surgery, *n* (%)	14,720 (83.4%)
	Rapid sequence induction, *n* (%)	1701 (9.6%)
Medical history	HTN, *n* (%)	3249 (18.4%)
	CHF, *n* (%)	206 (1.2%)
	CKD, *n* (%)	268 (1.5%)
	Atrial fibrillation, *n* (%)	527 (3.0%)
	Previous MI, *n* (%)	317 (1.8%)
	ACEi/ARB, *n* (%)	799 (4.5%)
	b-blockers, *n* (%)	1278 (7.2%)
ASA-PS	ASA-PS 1, *n* (%)	3442 (19.5%)
	ASA-PS 2, *n* (%)	6233 (35.3%)
	ASA-PS 3, *n* (%)	6789 (38.5%)
	ASA-PS 4, *n* (%)	1135 (6.4%)
	ASA-PS 5, *n* (%)	44 (0.2%)
Preinduction hemodynamic parameters	HR before, bpm, mean ± SD	80 ± 17
	MAP before, mmHg, mean ± SD	103 ± 18
	SAP before, mmHg, mean ± SD	143 ± 28
Induction drugs	Midazolam, mg/kg, mean ± SD	0.03 ± 0.01
	Fentanyl, mcg/kg, mean ± SD	1.94 ± 0.85
	Propofol, mg/kg, mean ± SD	1.98 ± 0.83
	Rocuronium, mg/kg, mean ± SD	0.68 ± 0.29
	Lidocaine, mg/kg, mean ± SD	1.04 ± 0.37
Post-induction anesthesia	MAC 1–10 min, mean ± SD	0.52 ± 0.23
	Isoflurane, *n* (%)	13,866 (78.6%)
	Sevoflurane, *n* (%)	3779 (21.4%)

A total of 21,309 adult patients who underwent non-obstetric, non-cardiac inpatient surgery under general anesthesia were initially screened. Exclusion criteria included patients arriving intubated (*n* = 713), those receiving vasoactive medications before and during induction (n = 1,380), and those with mechanical circulatory support prior to induction (*n* = 23). An additional 1,548 patients were excluded due to missing data. The final study cohort included 17,645 patients.

### MAP trajectories

**Fig. 2:**
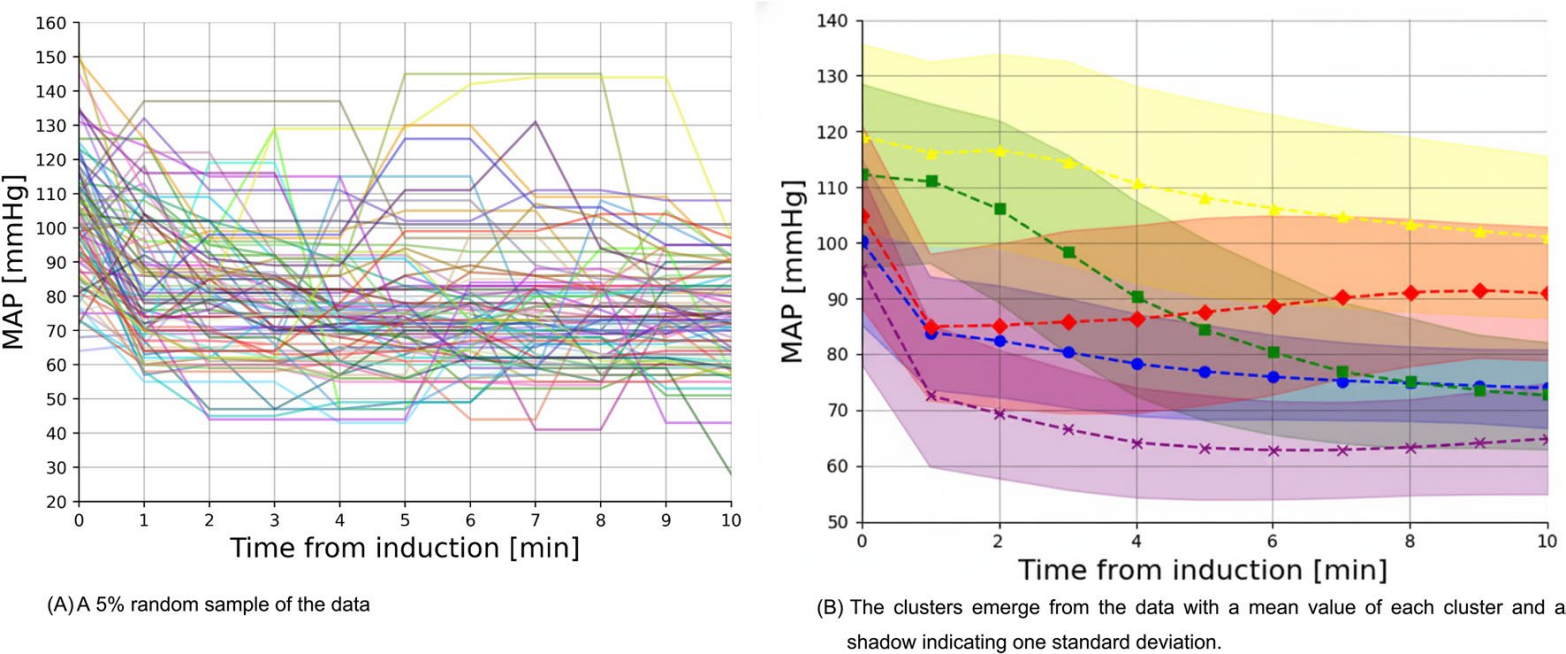
Mean blood pressure (MAP) trajectories 10 min after intravenous induction. Five blood pressure trajectories were identified. Time zero corresponds to the start of induction. Solid lines represent the mean observed blood pressure.

The analysis included a total of 176,450 time-stamped MAP measurements. The clustering method identified five distinct MAP trajectories (Fig. 2). The value of five distinct MAP trajectories is found using the elbow-point method following the Bayesian information criterion of 4097.14, 2719.39, 2405.17, 2212.98, 2140.24, 2122.70, 2156.07, 2147.96, 2189.55, and 2246.20 for a number of clusters that range from 1 to 10.

Five blood pressure trajectories were identified. Time zero corresponds to the start of induction. Solid lines represent the mean observed blood pressure, and shaded areas represent the 95% CI. (a) 5% random sample of the data. (b) The clusters emerge from the data.

We named these five clusters according to their hemodynamic profiles: Initial Decline—Plateau (5618, 31.8%) – the largest group, where patients initially experience a decrease in MAP but maintain stable blood pressure throughout the 10 min postinduction; Gradual Moderate Decline (3243, 18.4%) – this group shows a gradual decline in blood pressure postinduction, starting with higher pre-induction MAP values; Initial Decline—Recovery (1330, 7.5%) – the smallest group, where patients experience an initial drop in MAP followed by a gradual rise in blood pressure; Gradual Severe Decline (5218, 29.6%) – this group experiences the most significant drop in blood pressure after induction compared with other trajectories, with an absolute drop of around 40 mmHg; and Initial Decline—Low Plateau (2236, 12.7%) – these patients start with relatively low blood pressure compared with other groups, and after a substantial drop, they maintain this low level postinduction. A comparison of trajectory groups is shown in Table 2. In order to emphasize that MAP values become less correlated as the time between them increases, a Pearson heatmap of the MAP values over time is provided as Supplemental Fig. 1.

**Table 2:** Comparison between different clusters.

Parameter	Initial Decline—Plateau 5618 (31.8%)	Gradual Moderate Decline 3243 (18.4%)	Initial Decline—Recovery 1330 (7.5%)	Gradual Severe Decline 5218 (29.6%)	Initial Decline—Low Plateau 2236 (12.7%)	P value
Male, *n* (%)	3005 (53.5%)	1769 (54.5%)	716 (53.8%)	2763 (53.0%)	1184 (53.0%)	0.664
Elective surgery, *n* (%)	4703 (83.7%)	2674 (82.5%)	1108 (83.3%)	4391 (84.2%)	1844 (82.5%)	0.197
HTN, *n* (%)	750 (13.3%)	793 (24.5%)	469 (35.3%)	787 (15.1%)	450 (20.1%)	< 0.001
CHF, *n* (%)	58 (1.0%)	32 (1.0%)	33 (2.5%)	54 (1.0%)	29 (1.3%)	< 0.001
CKD, n (%)	68 (1.2%)	72 (2.2%)	42 (3.2%)	50 (1.0%)	36 (1.6%)	< 0.001
Atrial fibrillation, *n* (%)	181 (3.2%)	102 (3.1%)	34 (2.6%)	142 (2.7%)	68 (3.0%)	0.479
Previous MI, *n* (%)	80 (1.4%)	76 (2.3%)	42 (3.2%)	72 (1.4%)	47 (2.1%)	< 0.001
ACEi/ARB, *n* (%)	153 (2.7%)	189 (5.8%)	120 (9.0%)	222 (4.3%)	115 (5.1%)	< 0.001
b-blockers, *n* (%)	289 (5.1%)	293 (9.0%)	157 (11.8%)	374 (7.2%)	165 (7.4%)	< 0.001
Rapid sequence induction, *n* (%)	510 (9.1%)	381 (11.7%)	109 (8.2%)	554 (10.6%)	147 (6.6%)	< 0.001
ASA-PS 1, *n* (%)	1120 (19.9%)	655 (20.2%)	260 (19.5%)	986 (18.9%)	421 (18.8%)	0.530
ASA-PS 2, *n* (%)	1987 (35.4%)	1077 (33.2%)	482 (36.2%)	1897 (36.4%)	790 (35.3%)	
ASA-PS 3, *n* (%)	2150 (38.3%)	1262 (38.9%)	501 (37.7%)	1998 (38.3%)	878 (39.3%)	
ASA-PS 4, *n* (%)	346 (6.2%)	239 (7.4%)	87 (6.5%)	323 (6.2%)	140 (6.3%)	
ASA-PS 5, *n* (%)	14 (0.2%)	10 (0.3%)	0 (0.0%)	13 (0.2%)	7 (0.3%)	
Age, years, median [Q1-Q3]	48.2 [34.5—67.9]	48.4 [34.7–67.9]	48.9 [34.8–67.9]	47.9 [33.8–68.0]	48.7 [33.9–68.6]	0.542
BMI, kg/m2, mean ± SD	26.9 ± 6.0	26.9 ± 6.1	26.7 ± 5.9	26.9 ± 6.1	26.9 ± 6.2	0.769
MAC 1–10 min, mean ± SD	0.54 ± 0.23	0.53 ± 0.22	0.47 ± 0.24	0.53 ± 0.22	0.48 ± 0.24	< 0.001
HR before, bpm, mean ± SD	80 ± 17	80 ± 17	79 ± 17	80 ± 18	79 ± 17	0.059
MAP before, mmHg, mean ± SD	100 ± 15	112 ± 17	119 ± 17	95 ± 17	105 ± 17	< 0.001
SAP before, mmHg, mean ± SD	139 ± 23	157 ± 27	168 ± 28	132 ± 27	146 ± 27	< 0.001
Midazolam, mg/kg, mean ± SD	0.03 ± 0.01	0.03 ± 0.01	0.03 ± 0.02	0.03 ± 0.01	0.03 ± 0.01	< 0.001
Fentanyl, mcg/kg, mean ± SD	1.98 ± 0.85	1.94 ± 0.83	1.92 ± 0.90	1.91 ± 0.85	1.94 ± 0.87	0.005
Propofol, mg/kg, mean ± SD	2.07 ± 0.84	1.98 ± 0.85	1.92 ± 0.82	1.93 ± 0.82	1.93 ± 0.83	< 0.001
Rocuronium, mg/kg, mean ± SD	0.67 ± 0.28	0.71 ± 0.29]	0.73 ± 0.31	0.65 ± 0.28	0.68 ± 0.29	< 0.001
Lidocaine, mg/kg, mean ± SD	1.05 ± 0.37	1.07 ± 0.37	1.11 ± 0.36	0.98 ± 0.37	1.06 ± 0.40	< 0.001

BMI, body mass index; HTN, hypertension; CHF, congestive heart failure; CKD, chronic kidney disease; MI, myocardial infarction; ACEi/ARB, angiotensin-converting enzyme inhibitors/angiotensin II receptor blockers; HR, heart rate; bpm, beats per minute; MAP, mean arterial pressure; SAP, systolic arterial pressure; MAC, minimum alveolar concentration; mg, milligrams; mcg, micrograms; mg/kg, milligrams per kilogram; mcg/kg, micrograms per kilogram. Variables with a normal distribution were reported as mean ± standard deviation (SD), while non-normally distributed variables were reported as median and interquartile range (IQR), expressed as actual ranges (Q1–Q3), and number (percentage) for categorical variables.

## Discussion

In this large retrospective cohort of 17,645 adult patients, we employed a time-series clustering approach to characterize five distinct blood pressure trajectories during the initial 10 min following induction of general anesthesia. These trajectories—Initial Decline—Plateau, Gradual Moderate—Decline, Initial Decline—Recovery, Gradual Severe Decline, and Initial Decline—Low Plateau—underscore the heterogeneity in early postinduction hemodynamic profiles, beyond the binary notion of whether BP simply “drops or not.” Although the phenomenon of postinduction hypotension is widely recognized, it can manifest in substantially different temporal patterns. To our knowledge, this is the first large-scale study to apply unsupervised time-series clustering to characterize MAP behavior across a real-world surgical cohort.

Among the trajectories, the Gradual Severe Decline and the Initial Decline—Plateau were the largest groups, together comprising over 60% of patients. From a clinical standpoint, the Gradual Severe Decline group may be the most challenging because their rapid and sizable fall in MAP often occurs before clinicians can fully respond with fluid boluses or vasoactive agents. Even brief delays in recognizing or treating this precipitous drop could predispose patients to insufficient end-organ perfusion, especially if they have limited physiological reserve. Identifying trajectory-specific risk factors, such as comorbidities or baseline vascular tone, could help anticipate which patients are at greatest risk and enable proactive measures—such as lower induction agent doses or early vasopressor support^11^.

In comparing the various trajectories, several notable differences emerge between the groups, particularly with regard to the patient’s comorbidities and details of anesthesia induction (Table 2). Although statistically significant differences in anesthetic agent doses were observed across the trajectory groups, these differences are unlikely to have clinical significance. For example, the propofol dose in the Initial Decline—Recovery was lower (1.92 ± 0.82 mg) compared with other groups, with the highest dose observed among the Initial Decline—Plateau (2.07 ± 0.84 mg). However, these variations in anesthetic dosing do not seem to account for the distinct BP trajectory patterns in a clinically relevant manner. This finding supports the concept that inherent patient-specific physiology, including comorbid conditions like hypertension or the chronic use of antihypertensives (e.g., ACE inhibitors/ARBs), likely exerts a stronger influence on early postinduction BP trajectories than induction drug doses alone. Understanding these five patterns has practical relevance for anesthesiologists. For instance, a Gradual Moderate—Decline may allow slightly more time to mitigate hypotension with fluid administration or small-dose vasopressors, whereas an Gradual Severe Decline demands proactive preventive measures. Similarly, Initial Decline—Recovery group might initially appear hypotensive but recover spontaneously, raising questions about the optimal timing of interventions to avoid unnecessary overtreatment. In all cases, a trajectory-based perspective allows clinicians to move beyond a one-size-fits-all threshold and consider individualized, temporally informed management strategies.

Our study has several limitations. The most important limitation is the absence of time-resolved anesthetic dosing data. Due to the retrospective design, we were unable to determine detailed anesthetic drug administration patterns or clinicians’ real-time responses to blood pressure changes. For example, MAC values were recorded as averages over the 10-min postinduction period, reflecting early anesthetic maintenance; however, minute-by-minute MAC data were not available, precluding analysis of dynamic titration patterns. Detailed documentation of intra-induction fluid administration was also inconsistent. Given the potential influence of fluid therapy on blood pressure trends, this represents another unmeasured variable. Furthermore, patients who received vasoactive medications before or during the first 10 min after induction were excluded to minimize pharmacologic confounding and isolate intrinsic blood pressure responses. This approach, however, may have introduced selection bias by excluding patients at higher risk of hemodynamic instability, thereby limiting the generalizability of our findings to broader surgical populations.

Without these key data, it is impossible to determine whether the observed MAP trajectories primarily reflect intrinsic physiological responses to anesthesia induction, anesthetic dosing patterns, clinicians’ interventions, or a combination of these factors. Nevertheless, several considerations mitigate this uncertainty. In clinical practice, induction agents such as propofol are typically administered as brief boluses or short titrations, and subsequent adjustments in anesthetic depth through MAC modification occur gradually rather than abruptly. Moreover, within a 10-min window, there is limited opportunity to administer a clinically meaningful volume of fluid capable of substantially altering blood pressure. Taken together, these considerations support the view that the early postinduction MAP dynamics likely reflect intrinsic physiological behavior at least to some extent, rather than purely the effects of clinical actions.

Additional limitations include the single-centre design, which may restrict the external validity of our findings, and the potential for unmeasured confounding inherent in retrospective analyses. We cannot exclude the possibility that other unrecorded factors influenced blood pressure trajectories.

Overall, these limitations indicate that the present findings should be interpreted as descriptive and hypothesis-generating rather than causal or practice-changing. The primary contribution of this work is methodological: we introduce a reproducible time-series clustering framework that can be applied to larger, richer datasets with detailed clinical context, advancing data-driven understanding of intraoperative hemodynamics.

Future research should prospectively validate these trajectories in multicentre settings and examine their associations with postoperative outcomes such as myocardial injury, acute kidney injury, or mortality. In parallel, predictive modeling algorithms could be developed to classify patients into trajectory groups based on preoperative and early intraoperative variables. Once validated, such models could be integrated into electronic health record systems to support real-time, personalized risk stratification and guide tailored induction strategies. These steps will be essential for translating trajectory-based insights from observational analysis into actionable decision-support tools for individualized hemodynamic management.

## Supplementary Information

Below is the link to the electronic supplementary material.

Supplementary Material 1

Supplementary Material 2

Supplementary Material 3

## Data Availability

The datasets generated and/or analysed during the current study are not publicly available due to privacy reasons according to Israeli law, but are available from the corresponding author on reasonable request.

## Abbreviations

BMI: Body mass index
HTN: Hypertension
CHF: Congestive heart failure
CKD: Chronic kidney disease
MI: Myocardial infarction
ACEi/ARB: Angiotensin-converting enzyme inhibitors/angiotensin II receptor blockers
HR: Heart rate
bpm: Beats per minute
MAP: Mean arterial pressure
SAP: Systolic arterial pressure
MAC: Minimum alveolar concentration
mg: Milligrams
mcg: Micrograms
mg/kg: Milligrams per kilogram
mcg/kg: Micrograms per kilogram. Variables with a normal distribution were reported as mean ± standard deviation (SD), while non-normally distributed variables were reported as median and interquartile range (IQR), expressed as actual ranges (Q1–Q3), and number (percentage) for categorical variables

## Declarations

### Constent for publication

During the preparation of this work, the authors used “ChatGPT” (by OpenAI) for text and grammar correction. After using this tool, the authors reviewed and edited the content as needed and took full responsibility for the content of the publication.
