# Supplementary material for: A time-series clustering analysis of postinduction blood pressure trajectories
Source: Sci Rep. 2026 Jan 16;16:3745. doi: 10.1038/s41598-025-33740-x (PMC12852085; doi:10.1038/s41598-025-33740-x)

Supplementary Materials

Supplemental Table 1. Distribution of surgical procedures by department.

Operating department	Number of patients (%)
General surgery	6692 (37.93)
Orthopedic surgery	2836 (16.07)
Urology	1826 (10.35)
ENT	1426 (8.08)
Neurosurgery	1273 (7.21)
Hand surgery	983 (5.57)
Thoracic surgery	591 (3.35)
Plastic surgery	506 (2.87)
Ginecology	461 (2.61)
Oromaxillary surgery	357 (2.02)
Vascular surgery	572 (3.24)
Ophtalmology	122 (0.69)

Supplemental Figure 1. Pearson correlation matrix between the MAP time stamps.

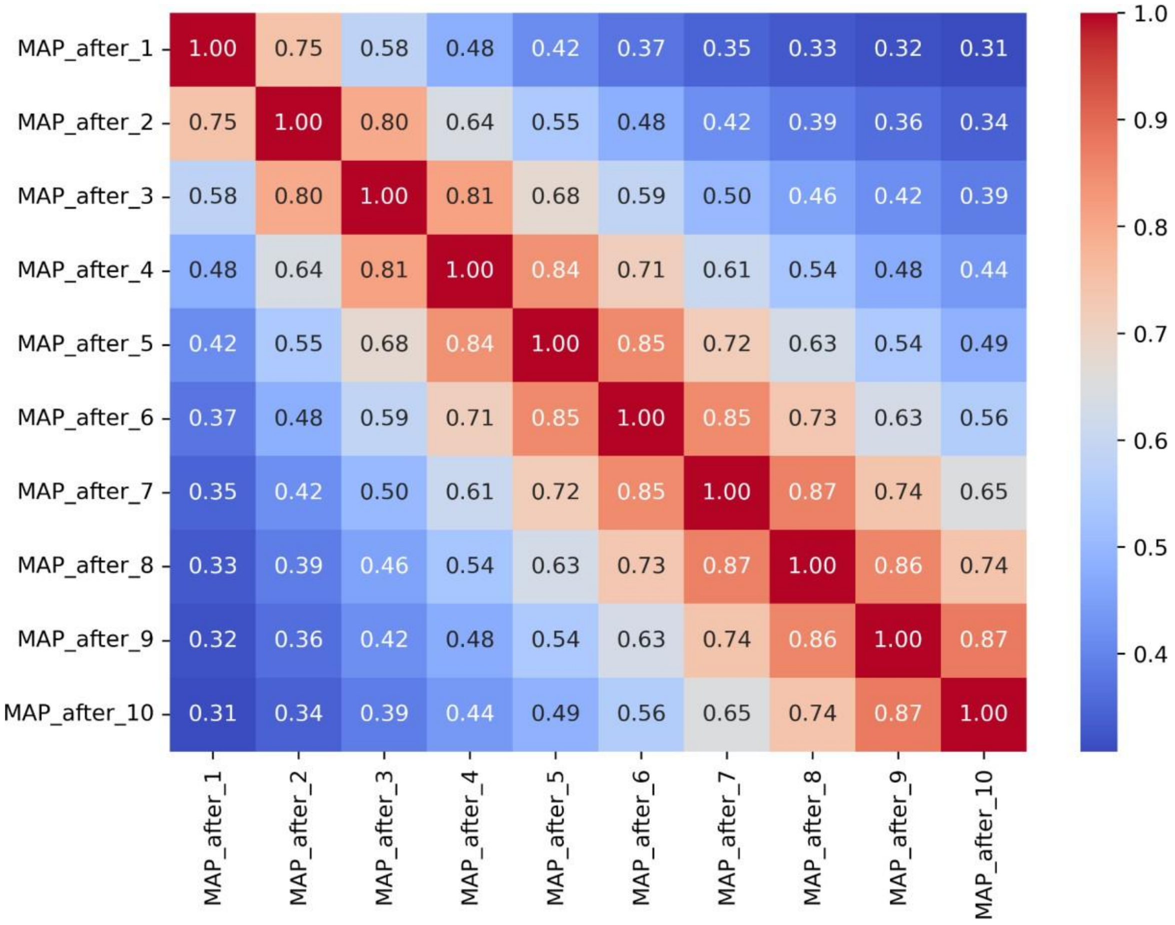

Supplement: Supplementary file 1 — Supplementary Material 1 [file 41598_2025_33740_MOESM1_ESM.pdf]
