# Supplementary material for: A time-series clustering analysis of postinduction blood pressure trajectories
Source: Sci Rep. 2026 Jan 16;16:3745. doi: 10.1038/s41598-025-33740-x (PMC12852085; doi:10.1038/s41598-025-33740-x)
Supplement: Supplementary file 2 — Supplementary Material 2 [file 41598_2025_33740_MOESM2_ESM.pdf]

Supplemental Table 1*: Independent variables used in developing the prediction model.*

Variables	Measurement timing	Missing values
Age	preoperative	0
Weight	preoperative	0
Height	preoperative	0
Body mass index	preoperative	0
Sex	preoperative	0
ASA score	preoperative	0
Elective surgery (Yes or No)	preoperative	0
Smoking history (Yes or No)	preoperative	967
Hypertension (Yes or No)	preoperative	0
Valvular heart disease (Yes or No)	preoperative	0
Congestive heart failure (Yes or No)	preoperative	0
Chronic kidney disease (Yes or No)	preoperative	0
Atrial fibrillation (Yes or No)	preoperative	0
Coronary artery disease (Yes or No)	preoperative	0
Myocardial infarction in the past (Yes or No)	preoperative	0
Diabetes mellitus (Yes or No)	preoperative	0
Peripheral vascular disease (Yes or No)	preoperative	0
Angiotensin-converting enzyme inhibitor or angiotensin receptor blocker medications (Yes or No)	preoperative	0
Calcium channel blocker medications (Yes or No)	preoperative	0
Beta-blocker medications (Yes or No)	preoperative	0
Diuretic medications (Yes or No)	preoperative	0
Thyroid replacement therapy (Yes or No)	preoperative	0
Selective serotonin reuptake inhibitor medications (Yes or No)	preoperative	0
Steroid medications (Yes or No)	preoperative	0
Rapid sequence induction (Yes or No)	induction	0

Endotracheal tube (Yes or No)	induction	0
Midazolam dosage (mg/kg)	induction	0
Fentanyl dosage (mg/kg)	induction	0
Propofol dosage (mg/kg)	induction	0
Etomidate dosage (mg/kg)	induction	0
Ketamine dosage (mg/kg)	induction	0
Rocuronium dosage (mg/kg)	induction	0
Succinylcholine dosage (mg/kg)	induction	0
Lidocaine dosage (mg/kg)	induction	0
Minimum alveolar concentration (MAC) at 1-10 minutes	after induction	0
Heart rate before induction	before induction	0
Mean arterial pressure before induction	before induction	0
Systolic arterial pressure before induction	before induction	0
